# Determinants of Pancreatic Size and Fat Content in the UK Biobank: Influence of Race, Genetic Variants, and Risk Factors

**DOI:** 10.1210/clinem/dgaf420

**Published:** 2025-07-19

**Authors:** Tanvi Kongara, Alexander Carruth, Prahlad Bhat, John Virostko

**Affiliations:** Department of Diagnostic Medicine, Dell Medical School, University of Texas at Austin, Austin, TX 78712, USA; Department of Diagnostic Medicine, Dell Medical School, University of Texas at Austin, Austin, TX 78712, USA; Department of Diagnostic Medicine, Dell Medical School, University of Texas at Austin, Austin, TX 78712, USA; Department of Diagnostic Medicine, Dell Medical School, University of Texas at Austin, Austin, TX 78712, USA; Oden Institute for Computational Engineering and Sciences, The University of Texas at Austin, Austin, TX 78712, USA; Livestrong Cancer Institutes, Dell Medical School, University of Texas at Austin, Austin, TX 78712, USA

**Keywords:** T1D, T2D, diabetes, PFF, fat fraction, genetic risk score

## Abstract

**Objective:**

Pancreas size and fat content differ in individuals with diabetes, but it is not clear how pancreas imaging features are impacted by race, genetics, and lifestyle factors.

**Methods:**

We performed cross-sectional analysis of 37 163 UK Biobank participants with pancreas imaging data to investigate racial and genetic influences on pancreas volume index (PVI) and pancreatic fat fraction (PFF). We further assessed the influence of prior glycemic status and lifestyle factors on pancreas size and composition after adjusting for sex and age.

**Results:**

White nondiabetic individuals had lower PVI compared to Asian participants (*P* < .0001), but higher than Black participants (*P* < .01), whereas PFF was highest in White participants. Similar trends were observed in participants with diagnosed diabetes. When stratified by glycemic status acquired 4 to 14 years before imaging, White and Asian participants exhibited significantly lower PVI and higher PFF with increased hemoglobin A1c. Higher polygenic risk scores for type 2 diabetes and hemoglobin A1c were associated with reduced PVI (ρ = −0.07, *P* < .0001; and ρ = −0.14, *P* < .0001, respectively) and elevated PFF (ρ = 0.07, *P* < .0001 and ρ = 0.18, *P* < .0001, respectively). In contrast, type 1 diabetes polygenic risk showed no significant association with any pancreas measures. Sedentary lifestyle behaviors associated with type 2 diabetes risk at study baseline correlated with smaller PVI and increased pancreas fat 4 to 14 years later.

**Conclusion:**

These findings highlight contributions of race, genetics, and behaviors to pancreatic morphology and fat deposition in glycemic dysregulation.

Although diabetes is largely defined by impaired glucose metabolism, variations in pancreas structure may contribute to differences in insulin production and β-cell function (1, 2). Of note, small pancreas size (3, 4) and increased fat content (5) may predict individuals at risk for developing diabetes. The disproportionate impact of diabetes on minority groups and individuals from lower socioeconomic backgrounds may arise from both social and structural determinants of disease susceptibility (6) as well as underlying metabolic differences (7). Emerging evidence indicates that the relationship between pancreatic fat accumulation and β-cell dysfunction may differ across racial and ethnic groups (8), suggesting potential biological or environmental modifiers of disease progression.

As most imaging studies of the pancreas in individuals with diabetes have been performed in small, homogenous study populations, it is not clear if pancreas size differs across race. Furthermore, it is not known how pancreas features interact with modifiable lifestyle factors and genetic underpinnings of diabetes risk.

This study seeks to clarify the impact of race, genetics, and lifestyle factors on pancreas size and fat fraction. We conducted a cross-sectional analysis of pancreas magnetic resonance imaging (MRI) data from the UK Biobank, a large-scale, population-based observational study. Our findings suggest that race, genetics, and modifiable risk factors can influence pancreas size and fat content.

## Materials and Methods

### Data Sources and Study Participants

Participants in the UK Biobank, an observational study of subjects in the United Kingdom aged 40 to 69 years, who received abdominal MRI were analyzed (n = 37 163). Participant characteristics stratified by race are shown in Table 1. Data were retrieved from the UK Biobank on April 16, 2024. For participants with multiple imaging visits, only data from the first abdominal MRI scan were used to avoid duplication. Abdominal MRI was performed using a Siemens Aera 1.5T scanner (Siemens, Erlangen, Germany) and included a high-resolution acquisition to calculate pancreas volume and multiecho acquisition to assess pancreatic fat fraction (PFF). The pancreas was automatically segmented using a U-net for calculation of pancreas volume and PFF, as previously described (9). Whole-genome sequencing was performed on UK Biobank participants and used to calculate polygenic risk scores (PRS) for type 1 diabetes (T1D), type 2 diabetes (T2D), and hemoglobin A1c (HbA1c) (10). The UK Biobank also captured demographic, health, physical, biomarker, and lifestyle variables from participants. We used the following data fields from the database at the time of imaging: diabetes diagnosed by a doctor at imaging (Field ID 2443), pancreas volume (Field ID 21087), pancreas fat fraction (Field ID 2190), weight at imaging (Field ID 21002), and ethnic background (Field ID 21000). We also used the following fields from baseline (4-14 years before imaging): HbA1c at baseline (Field ID 30750), time spent watching television (Field ID 1070), time spent using computer (Field ID 1080), number of days/week of vigorous physical activity (Field ID 904), and sleep duration (Field ID 1160).

**Table 1.** Study participant demographics and clinical characteristics

	Asian (N = 504)	Black (N = 235)	White (N = 36 424)
Age at baseline assessment			
Mean (SD)	51.6 (7.82)	49.8 (6.87)	55.0 (7.45)
Median [Min, Max]	51.0 [40.0, 70.0]	49.0 [40.0, 69.0]	56.0 [40.0, 70.0]
Age at MRI			
Mean (SD)	60.1 (7.97)	58.3 (6.93)	63.7 (7.52)
Median [Min, Max]	59.0 [44.0, 79.0]	58.0 [47.0, 78.0]	64.0 [45.0, 82.0]
Years between baseline and MRI			
Mean (SD)	8.43 (1.78)	8.54 (1.71)	8.73 (1.74)
Median [Min, Max]	9.00 [4.00, 13.0]	9.00 [4.00, 12.0]	9.00 [4.00, 14.0]
BMI			
Mean (SD)	25.0 (3.76)	28.2 (5.30)	25.9 (4.28)
Median [Min, Max]	24.5 [16.7, 41.2]	27.4 [19.5, 62.1]	25.3 [12.8, 64.9]
HbA1c (%)			
Mean (SD)	5.57 (0.600)	5.62 (0.990)	5.35 (0.470)
Median [Min, Max]	5.50 [3.76, 9.18]	5.54 [4.13, 15.7]	5.32 [3.55, 11.6]
Missing	44 (8.7%)	52 (22.1%)	2588 (7.1%)
Diagnosed diabetes			
No diabetes	422 (83.7%)	198 (84.3%)	34 424 (94.5%)
Diabetes	82 (16.3%)	36 (15.3%)	1998 (5.5%)
Missing	0 (0%)	1 (0.4%)	2 (0.0%)
Sex			
Female	206 (40.9%)	128 (54.5%)	18 877 (51.8%)
Male	298 (59.1%)	107 (45.5%)	17 547 (48.2%)

Abbreviations: BMI, body mass index; HbA1c, hemoglobin A1c; MRI, magnetic resonance imaging.

### Derived Variables

We derived several variables from UK Biobank data fields to facilitate analysis and conform with standard usage. Pancreas volume was normalized by body weight to yield pancreas volume index (PVI) (11). HbA1c levels were categorized into normoglycemic (<5.7%), prediabetes (5.7-6.4%), and diabetes (>6.5%) groups. To capture potential nonlinear associations and characterize individuals at the extremes of the distribution, PRS were categorized into percentile-based bins, with narrower groups at the tails. For the lifestyle variables, categories with similar characteristics or low frequencies were combined. This recategorization consisted of vigorous activity (<2 times per week vs ≥2 times per week), time spent watching television (≤4 hours per week vs >4 hours per week), time spent using computer (≤4 hours per week vs >4 hours per week), and sleep duration (1 to 6 hours, 7 to 8 hours, > 8 hours).

### Statistical Analysis

All analyses were performed using R (version 4.3.1). Comparisons of 2 groups used a Wilcoxon rank-sum test. Comparison of more than 2 groups employed ANOVA followed by pairwise Wilcoxon rank-sum test with Bonferroni correction. To account for sex- and age-related differences in pancreas size and fat fraction (12), we performed multivariable linear regression to examine the association between the pancreas image feature (pancreas volume, PVI, PFF) and primary independent variable of interest (race, HbA1c, lifestyle factors) adjusting for sex and age at imaging. Analysis of trends was assessed using Spearman rank correlation coefficient. A *P* value < .05 was considered statistically significant for all statistical testing. *P* values are reported to 3 decimal places, with values less than .0001 reported as *P* < .0001.

## Results

**Figure 1.**
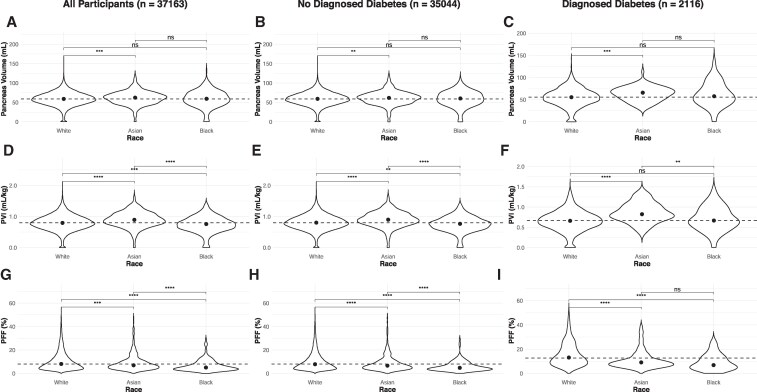
Pancreas volume is (A) significantly higher in Asian than White UK Biobank participants including (B) participants without diagnosed diabetes and (C) participants with diagnosed diabetes. When pancreas volume is normalized by body weight to yield a pancreas volume index (PVI), (D) PVI is highest in Asian, then White, then Black participants. This trend holds true for (E) participants without diagnosed diabetes and (F) participants with diagnosed diabetes. Pancreas fat fraction (PFF) is (G) highest for White, then Asian, then Black participants. This pattern is similar in subgroups (H) without diagnosed diabetes and (I) with diagnosed diabetes. Asterisks indicate significance levels (*P < .05, **P < .01, ***P < .001, ****P < .0001; ns = not significant).

In the first analysis (Fig. 1), we examined the relationship between pancreas volume, PVI, and PFF across 3 racial groups (White, Asian, and Black participants) stratified into participants with and without diagnosed diabetes. White participants without diabetes had a lower PVI than Asian participants (*P* < .0001), but higher PVI than Black participants (*P* < .01). PFF was highest in White participants, followed by Asian and then Black participants. The number of participants with diagnosed diabetes was lower but similarly demonstrated lower PVI in White than Asian participants (*P* < .0001). Participants with diagnosed exhibited higher PFF in White participants than Asian and Black participants. When adjusted for age and sex, PVI was still significantly higher in Asian participants and lower in Black participants compared with White participants (*P* < .0001, both). PFF also shows a similar trend when adjusted for age and sex: PVI is significantly higher in White participants compared with Asian (*P* < .001) or Black (*P* < .0001) participants.

**Figure 2.**
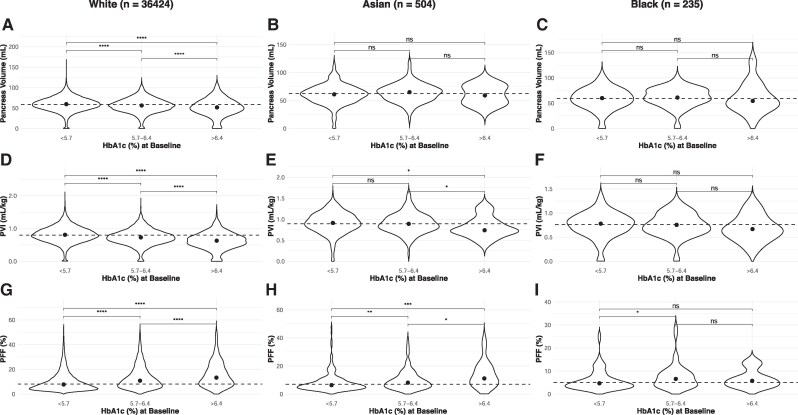
Pancreas volume (A) is smaller with higher HbA1c measured years earlier in White participants. Significant trends between pancreas volume and prior HbA1c were not detected in smaller cohorts of (B) Asian or (C) Black participants. PVI is lower with higher prior HbA1c in (D) White and (E) Asian participants but is not significantly associated in (F) Black participants. PFF correlates with prior HbA1c in (G) White, (H) Asian, and (I) Black participants. Asterisks indicate significance levels (*P < .05, **P < .01, ***P < .001, ****P < .0001; ns = not significant).

We next sought to determine how pancreas volume and fat content correlated with prior dysglycemia across racial groups. We assessed pancreas volume, PVI, and PFF as a function of glycemic status at study baseline (normoglycemic, prediabetes, and diabetes) in Asian, Black, and White participants (Fig. 2). In White participants, pancreas volume and PVI decreased and PFF increased with rising HbA1c measured at study baseline (*P* < .0001, all comparisons). These relationships remained significant when adjusted for age and sex. Asian participants demonstrated a significant decrease in and an increase in with increasing HbA1c. After adjusting for age and sex, Asian participants with diabetes had a higher PFF than normoglycemic participants (*P* = .008). Among a smaller cohort of Black participants, pancreas volume and PVI were not significantly different between any glycemic status groups. In Black participants, PFF was significantly higher (*P* < .05) in the prediabetes group than the normoglycemic group, but this relationship was not significant after adjusting for age and sex.

**Figure 3.**
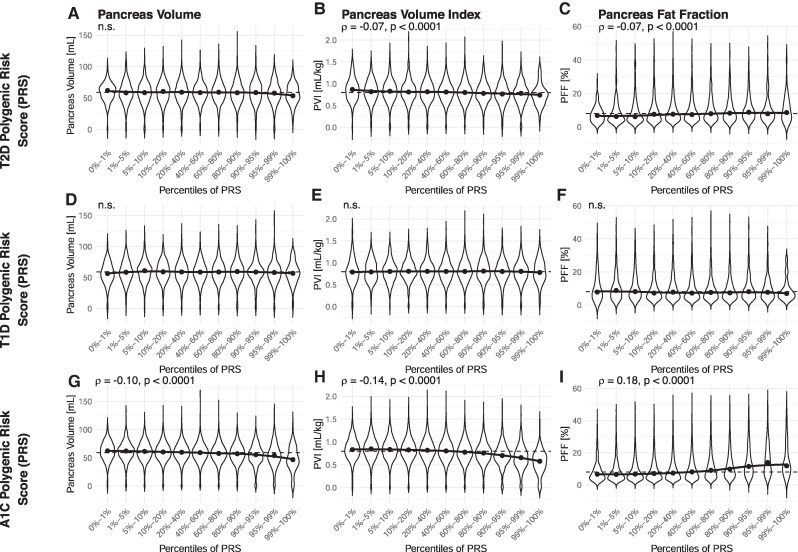
Polygenic risk scores (PRS) for type 2 diabetes (T2D) did not correlate with (A) pancreas volume, were negatively correlated with (B) PVI, and positively correlated with (C) PFF. In contrast, PRS for type 1 diabetes (T1D) did not correlate with (D) pancreas volume, (E) PVI, or (F) PFF. PRS for HbA1c were negatively correlated with (G) pancreas volume and (H) PVI, but (I) positively correlated with PFF. The solid line shows a localized regression through the median value for each PRS percentile, whereas the dashed line displays the median value across all PRS percentiles.

**Figure 4.**
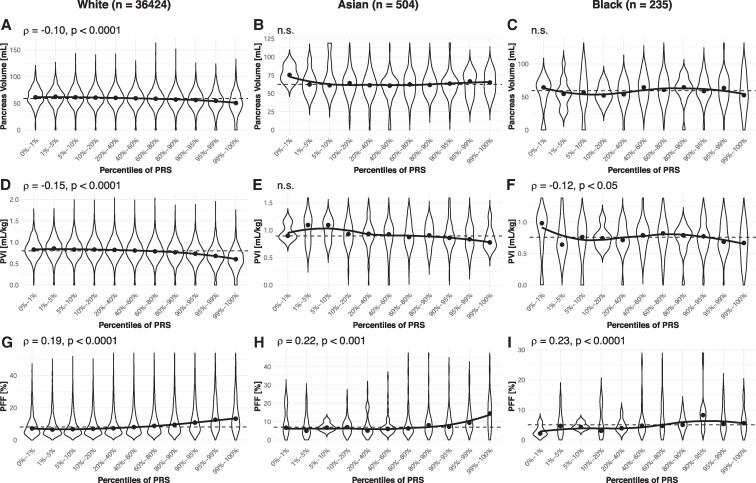
Pancreas volume negatively correlated with HbA1c PRS in (A) White participants, but not smaller cohorts of (B) Asian or (C) Black participants. PVI negatively correlated with HbA1c PRS in (D) White and (F) Black participants, but not a smaller cohort of (E) Asian participants. PFF correlated with HbA1c PRS in (G) White (H) Asian, and (I) Black participants. The solid line shows a localized regression through the median value for each PRS percentile, whereas the dashed line displays the median value across all PRS percentiles.

Given the influence of race on pancreas size and fat content, we examined the genetic contribution to pancreas features. PVI declined and PFF increased in individuals with higher T2D PRS (Fig. 3, ρ = −0.07, *P* < .0001 and ρ = 0.07, *P* < .0001, respectively). Similarly, PVI declined and PFF increased in individuals with higher HbA1c PRS (Fig. 3, ρ = −0.14, *P* < .0001 and ρ = 0.18, *P* < .0001, respectively). In contrast, we did not detect any significant correlation between T1D PRS and pancreas volume, PVI, or PFF. These results were consistent across men and women. To assess potential racial differences in genetic risk effects, we further stratified HbA1c PRS associations by race (Fig. 4) because this PRS was most strongly associated with differences in pancreas size and composition. Among White participants, higher HbA1c PRS percentiles were associated with a lower pancreas volume (ρ = −0.10, *P* < .0001) and PVI (ρ = −0.15, *P* < .0001), and a higher pancreas fat fraction (ρ = 0.19, *P* < .0001). Pancreas size trends were less consistent in smaller cohorts of Asian and Black participants, where only PVI in Black participants had a significant correlation with HbA1c (ρ = −0.12, *P* < .05). However, PFF was positively correlated with HbA1c PRS percentiles in both Black (ρ = 0.23, *P* < .0001) and Asian cohorts (ρ = 0.22, *P* < .01). When stratified for sex, all associations between HbA1c PRS percentiles and pancreas features remained significant (*P* < .0001) in both male and female White participants. PFF significantly correlated (*P* < .05) with HbA1c PRS percentiles in Asian men and women and Black women, but not Black men (*P* = .4).

**Figure 5.**
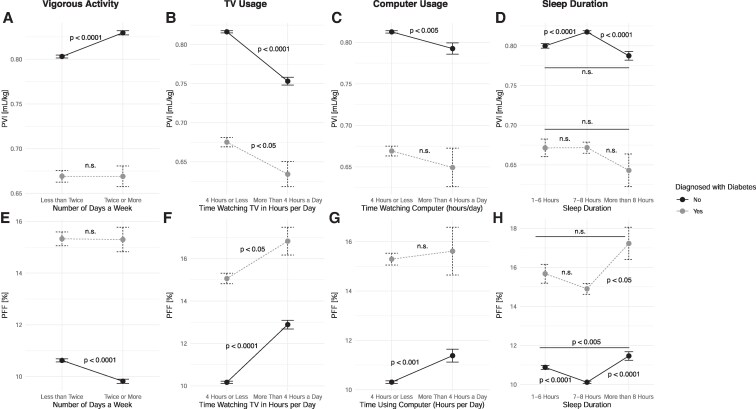
(A) PVI was higher in nondiabetic individuals, but not individuals with diabetes, with higher levels of physical activity assessed years prior to imaging. Participants who (B) watched more than 4 hours of television daily or (C) used a computer for more than 4 hours per had significantly lower PVI in participants without diabetes. (D) Nondiabetic participants who slept 7 to 8 hours per night had a significant higher PVI compared to those who slept 1 to 6 hours or those who slept more than 8 hours. (E) Nondiabetic participants who engaged in vigorous physical activity more than twice per week had significantly lower PFF compared to those who exercised less. Similarly, (F) watching television or (G) using a computer for more than 4 hours per day was associated with a higher PFF in nondiabetic individuals. (H) Nondiabetic individuals who slept 7 to 8 hours per night had significantly lower PFF compared to both shorter and longer sleepers. Individuals with diabetes who slept 7 to 8 hours per night had lower PFF than those who slept more than 8 hours. All *P* values were adjusted for sex and age at imaging.

Last, we examined the relationship between lifestyle factors known to confer diabetes risk surveyed at study baseline and pancreas measures performed 4 to 14 years later. All analyses were adjusted for sex and age at imaging. A significantly higher PVI was observed in participants who exercised more than twice per week compared to those who exercised less than twice per week (Fig. 5A) in participants not diagnosed with diabetes (*P* < .0001), but not in participants with diabetes (*P* = .95). Participants who watched more than 4 hours of television daily (Fig. 5B) had lower PVI in both participants with diabetes (*P* < .05) and those without diabetes (*P* < .0001). Participants who used a computer for more than 4 hours per day (Fig. 5C) had significantly lower PVI in participants without diabetes (*P* < .005), but not those with diabetes. Participants without diabetes who slept 7 to 8 hours per night (Fig. 5D) had a significant higher PVI compared to those who slept 1 to 6 hours (*P* < .0001) or those who slept more than 8 hours (*P* < .0001). Participants without diabetes did not exhibit any relationships between PVI and sleep per night. The relationship between lifestyle factors and PFF demonstrated higher fat content associated with behaviors associated with development of diabetes. Participants who engaged in vigorous physical activity more than twice per week had significantly lower PFF compared to those who exercised less frequently (Fig. 5E), but only in those without a diabetes diagnosis (*P* < .0001). Watching television (Fig. 5F) or using a computer (Fig. 5G) for more than 4 hours per day was associated with a higher PFF in nondiabetic individuals (*P* < .001, both). A weaker association between PFF and television usage was observed in those with diabetes (*P* < .05), but there was no significant association with computer usage. PFF also varied by sleep behavior in nondiabetic individuals (Fig. 5H): individuals who slept 7 to 8 hours per night had significantly lower PFF compared to both shorter (*P* < .0001) and longer sleepers (*P* < .005). Individuals diagnosed with diabetes who slept more than 8 hours had higher PFF than those who slept 7 to 8 hours (*P* < .05). Taken together, these findings suggest that sedentary behavior and irregular sleep contribute to increased pancreatic fat later in life, particularly in those without diagnosed diabetes at imaging.

## Discussion

In this study, we examined the influence of race, genetics, and prior dysglycemia and lifestyle factors on pancreas size and composition. Leveraging the large-scale UK Biobank cohort, we demonstrate that pancreas measurements are race dependent: Asian participants have the largest pancreas relative to weight, followed by White participants, and then Black participants. Pancreas fat content is similarly influenced by race with Whites having the highest fat content, followed by Asians, and then Black participants. We further demonstrate that smaller relative pancreas size and higher pancreas fat content is associated with higher prior HbA1c across racial groups. Genetic risk scores for T2D and HbA1c, but not T1D, are associated with smaller pancreas size and increased pancreatic fat content. Finally, we show that lifestyle factors associated with T2D influence pancreas size and fat content measured years later.

Our findings suggest that although smaller relative pancreas size and increased pancreatic fat consistently are associated with elevated HbA1c across all races, absolute thresholds should be interpreted with caution and may require adjustment for race. Our study found that Asian participants have a larger pancreas volume and PVI compared to White or Black participants. This contrasts with a previous study of Korean adults that found smaller pancreas volume than White participants (13). This may in part reflect differences in the demographics of the UK Biobank, in which the Asian population is primarily of South Asian descent (14). Moreover, the UK Biobank cohort includes both immigrants and individuals with immigrant parents, who may experience changes in diet, lifestyle, and physical activity. Pancreas fat fraction measurements were highest in White participants, followed by Asian and then Black participants. Prior studies of young (15) and middle-aged (16) adults also found lower pancreas fat content in Black participants. In general, smaller pancreas size and increased pancreas fat content are associated with diabetes. However, given the higher prevalence of diabetes in Black and Asian vs White populations in the United Kingdom (17), there may be different relationships between pancreas size and fat and diabetes risk across racial groups. Genetic risk scores for T2D and HbA1c, but not T1D, are associated with lower pancreas size and increased fat fraction, in agreement with a prior study using Mendelian randomization (18). These trends between pancreas size/composition and genetic risk scores appear similar across races. Importantly, although PRS can underperform across ethnic groups, UK Biobank PRS were developed while maximizing representation of non-European ancestries and validated across racial groups (10). Last, sedentary behavior and irregular sleep was associated with decreased PVI and increased PFF measured 4 to 14 years later. The strongest lifestyle correlations were observed in individuals without diabetes diagnosed at imaging, where regular exercise, moderate screen time, and healthy sleep habits were associated with reduced pancreatic fat content. This highlights a potential early intervention window, where modifiable behaviors could slow or reverse pancreas fat accumulation before diabetes onset. In contrast, these associations were diminished or absent in those already diagnosed with diabetes, suggesting that pancreatic atrophy and fat deposition may become less responsive to behavioral factors once metabolic dysfunction is established. We note that other lifestyle factors, such as smoking, alcohol use, and other sedentary behaviors, may affect pancreas size and fat content and should be explored in future work.

This study has several limitations related to the use of UK Biobank data. First, the UK Biobank cohort is not fully representative of the general population; participants tend to be healthier, older, and more socioeconomically advantaged than the broader population, which may limit generalizability. Second, individuals from racial and ethnic minority groups are underrepresented. Indeed, the White population in our study was 2 orders of magnitude larger than the Asian and Black populations, which limits our statistical power in these groups. Third, HbA1c and lifestyle factors were measured at baseline, but pancreas imaging was performed 4 to 14 years later. HbA1c likely increased over this period, and lifestyle factors may have changed. Our correlations thus demonstrate how earlier dysglycemia and sedentary behavior affects the pancreas later in life. We grouped participants with T1D and T2D in this study but note that pancreas image features may diverge between these populations (2). Because this study was considered exploratory analysis, *P* values were not adjusted for multiple comparisons. Thus, these findings require confirmation in independent cohorts.

In conclusion, this study highlights significant differences in pancreas size and composition based on race, dysglycemia, genetics, and lifestyle factors. These findings show the potential for pancreatic imaging in risk stratification for patients with diabetes, emphasizing the value in identifying modifiable variables to ultimately improve pancreatic health and minimize diabetes risk.

## Data Availability

This research was conducted using data from the UK Biobank under Application Number 79758. UK Biobank data are not publicly available due to participant confidentiality and data sharing agreements. However, researchers may apply for access to the data through the UK Biobank Access Management System. The derived data supporting the findings of this study are available from the authors upon reasonable request and with appropriate UK Biobank approvals.

## Abbreviations

HbA1c: hemoglobin A1c
MRI: magnetic resonance imaging
PFF: pancreatic fat fraction
PRS: polygenic risk score
PVI: pancreas volume index
T1D: type 1 diabetes
T2D: type 2 diabetes
