# Correction to: “Determinants of Pancreatic Size and Fat Content in the UK Biobank: Influence of Race, Genetic Variants, and Risk Factors”

**DOI:** 10.1210/clinem/dgaf518

**Published:** 2025-09-18

In the above-named article by Kongara T, Carruth A, Bhat P, and Virostko J (*J Clin Endocrinol Metab*. 2025; doi: 10.1210/clinem/dgaf420) there was an error in the Funding section.

In the originally published article, in the Funding section, the incorrect grant number “HD11556” was listed. In the corrected article, the correct grant number “HD115565” is listed and the sentence now reads, “Financial support was graciously provided by the National Institutes of Health (DK129979 and HD115565) and Breakthrough T1D (formerly JDRF) (1-INO-2023-1340-A-N).”

The article has been corrected online.

doi: 10.1210/clinem/dgaf420

