# Correction to: “Determinants of Pancreatic Size and Fat Content in the UK Biobank: Influence of Race, Genetic Variants, and Risk Factors”

**DOI:** 10.1210/clinem/dgag040

**Published:** 2026-02-09

In the above-named article by Kongara T, Carruth A, Bhat P, and Virostko J (*J Clin Endocrinol Metab*. 2025; 110(12): e3945-e3951; doi: 10.1210/clinem/dgaf420) there were errors in the Materials and Methods section and the Results section.

In the originally published article, in the Materials and Methods section, under the subheading “Derived Variables,” the last sentence read as, “This recategorization consisted of vigorous activity (<2 times per week vs ≥2 times per week), time spent watching television (≤4 hours per week vs >4 hours per week), time spent using computer (≤4 hours per week vs >4 hours per week), and sleep duration (1 to 6 hours, 7 to 8 hours, > 8 hours).” In the corrected article, “time spent watching television (≤4 hours per week vs >4 hours per week), time spent using computer (≤4 hours per week vs >4 hours per week)” was updated to “time spent watching television (≤4 hours per day vs >4 hours per day), time spent using computer (≤4 hours per day vs >4 hours per day),” and the sentence now reads, “This recategorization consisted of vigorous activity (<2 times per week vs ≥2 times per week), time spent watching television (≤4 hours per day vs >4 hours per day), time spent using computer (≤4 hours per day vs >4 hours per day), and sleep duration (1 to 6 hours, 7 to 8 hours, > 8 hours).”

In the originally published article, in the Results section, in the first paragraph, the sentence “PFF also shows a similar trend when adjusted for age and sex: PVI is significantly higher in White participants compared with Asian (P < .001) or Black (P < .0001) participants” incorrectly refers to “PVI.” In the corrected article, the sentence reads, “PFF also shows a similar trend when adjusted for age and sex: PFF is significantly higher in White participants compared with Asian (P < .001) or Black (P < .0001) participants.”

In the originally published article, in the Results section, in the fourth paragraph, the sentence “Participants without diabetes did not exhibit any relationships between PVI and sleep per night” incorrectly used the word “without” instead of “with.” In the corrected article, the sentence now reads, “Participants with diabetes did not exhibit any relationships between PVI and sleep per night.”

The article has been corrected online.

doi: 10.1210/clinem/dgaf420

